# DNA methylation meets lineage tracing: History, recent progress, and future directions

**DOI:** 10.1002/qub2.70017

**Published:** 2025-09-21

**Authors:** Ruijiang Fu, Mengyang Chen, Shou‐Wen Wang

**Affiliations:** ^1^ Westlake Laboratory of Life Sciences and Biomedicine Hangzhou Zhejiang China; ^2^ School of Life Sciences Westlake University Hangzhou Zhejiang China; ^3^ Department of Physics School of Science Westlake University Hangzhou Zhejiang China; ^4^ Center for Interdisciplinary Studies School of Science Westlake University Hangzhou Zhejiang China

**Keywords:** DNA methylation, lineage tracing, single‐cell multi‐omics

## Abstract

Lineage tracing techniques have been developed rapidly in the past decades by employing new genetic engineering tools. However, due to their invasive nature, these are difficult to apply to humans. Although endogenous DNA mutations can be used for in vivo lineage tracing in humans, their extremely low mutation rate presents substantial technical challenges. Epimutations on DNA methylation happen at a rate of about 0.001 per CpG site per division. Such rich and stable information enables high‐resolution, noninvasive lineage tracing in humans, as recently achieved with both MethylTree and EPI‐Clone. MethylTree is a computational innovation that accurately predicts cell lineages from single‐cell DNA methylation data, be it genome‐wide or targeted. EPI‐Clone is a targeted approach that requires careful CpG panel selection for specific tissues, which has been validated in blood. In this review, we present an overview of related historical studies, discuss the development of both MethylTree and EPI‐Clone, and compare these two approaches. Although EPI‐Clone is more scalable and cheaper, MethylTree has a higher resolution and works directly across different tissues. We demonstrate here that MethylTree also works well with EPI‐Clone data, thus providing a unified solution for epimutation‐based lineage tracing. Finally, we highlight the advantages of epimutation‐based lineage tracing, discuss future directions for tool development, and touch on considerations in biological applications. Epimutation‐based lineage tracing opens up an exciting avenue for noninvasive lineage tracing in humans across many biological processes.

## 1 INTRODUCTION

Lineage tracing is a technique that utilizes distinct and heritable markers to record cell division histories. It is a powerful tool to study development, tissue homeostasis, aging, and disease progression. Since Sulston et al. obtained the division histories of all cells in *C*.*elegans* in 1983 using live imaging [1], lineage tracing tools have progressed substantially over the last few decades. Importantly, DNA‐based labeling techniques can be combined with next‐generation sequencing for high‐throughput lineage tracing [2, 3, 4, 5, 6]. Among these developments, we generated a DARLIN mouse model that can produce 10^18^ unique barcodes to label individual cells and achieve efficient single‐cell readout of these lineage barcodes [7, 8]. Coupled with computational tools such as CoSpar [9, 10], these approaches have enabled a systematic study of cell fate decisions. However, most of these tools require genetic engineering, which is not applicable to human studies. In this review, we mainly focus on an overview of noninvasive lineage tracing based on endogenous markers.

Somatic DNA mutations have been shown to be reliable for lineage tracing in humans [2, 11]. However, these mutations are extremely rare in normal cells. With a mutation rate of just 10^−9^ per base pair per division, somatic mutations occur approximately only once per cell division [11, 12]. To detect such rare mutations, a cell needs to be expanded clonally in vitro, followed by deep whole‐genome sequencing [13, 14, 15]. This approach is not compatible with nondividing cells, does not provide a single‐cell multi‐omic readout, and costs about $500 per cell in total for cell culture, library construction, and sequencing.

Mutations in mitochondrial DNA (mtDNA) are an alternative for lineage tracing in humans [2, 16]. Although identifying mtDNA mutations is easier and cheaper experimentally, these mutations may not reliably track cell lineages. Because each cell contains hundreds of copies of mitochondrial DNA, the inheritance of an mtDNA mutation may suffer from neutral drift or undergo both positive and negative selection. Previous studies show that only a subset of mtDNA mutations could track cell lineages [17, 18]. However, it is challenging to know in advance which mutations would work. Furthermore, recent simulation and experimental data show that, due to neutral drift, mtDNA mutations work better for systems with massive clonal expansion, such as cancer, but perform poorly for systems with limited expansion [19]. Finally, mtDNA profiling still suffers from excessive data noises that need to be cleaned carefully to avoid erroneous biological conclusions [20]. Given the limitations of mutation‐based lineage tracing, a better approach is needed that utilizes other endogenous markers.

## 2 DNA METHYLATION FOR LINEAGE TRACING: A HISTORICAL PERSPECTIVE

**FIGURE 1:**
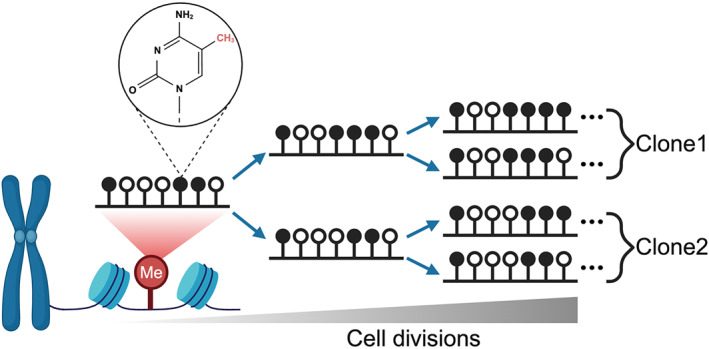
Schematic of the principle of DNA methylation‐based lineage tracing. The accumulation of epimutations on DNA methylation can be used to resolve cell lineages retrospectively.

Epigenetics regulates gene expression through chemical modifications or chromatin structure changes without altering the DNA sequence [21]. In mammals, DNA methylation primarily occurs at CpG sites in the genome, where the cytosine (C) can be modified into 5‐methylcytosine (5mC) and stably inherited during cell division (Figure 1) [22]. Here, methyltransferase DNMT1 is responsible for maintaining DNA methylation after DNA replication [23, 24], DNMT3A/B mediate de novo methylation [25], and TET enzymes enable demethylation on methylated CpG sites [26, 27]. The maintenance of DNA methylation is not entirely faithful over cell divisions. Early studies estimate the epimutation rate to be about 0.001 per CpG site per division [28, 29]. Given about 29 million CpG sites in the human genome, each cell division can accumulate tens of thousands of stochastic methylation changes. Once these epimutations occur, they can remain stable for hundreds of subsequent divisions. Therefore, DNA methylation could provide rich and stable information for high‐resolution reconstruction of cell lineages (Figure 1).

**FIGURE 2:**
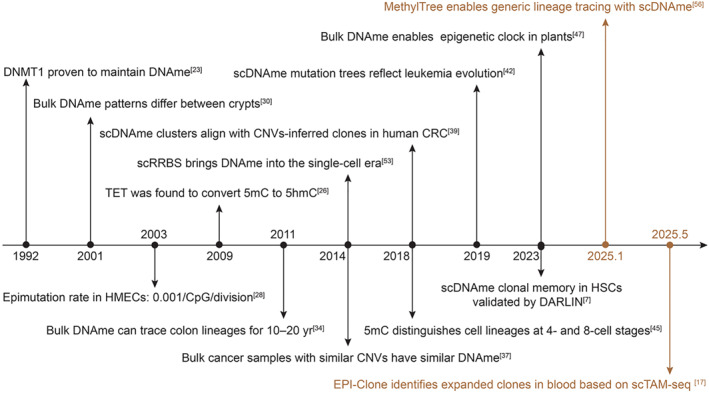
Timeline of developments related to DNA methylation‐based lineage tracing. This includes (1) studies revealing the inheritance of DNA methylation and the existence of epimutations; (2) studies showing that DNA methylation could record clonal information in the colon, cancer, early embryo, plants, and hematopoietic system; and (3) MethylTree and EPI‐Clone (brown). CRC, colorectal cancer; DNAme, DNA methylation; HMEC, normal human mammary epithelial cells; scDNAme, single‐cell DNA methylation.

In fact, several studies have suggested that DNA methylation could record clonal memories. The first insight came from the study of stem cell dynamics in the colon (Figure 2). In 2001, Yatabe et al. found that bulk DNA methylation patterns from cells within the same crypt were more similar than those from other crypts [30]. They also proposed that the heterogeneous DNA methylation patterns within the same crypt could arise from the existence of multiple stem cells in the crypt. Subsequent research by Kim et al. explained that clonal fixation within a crypt, driven by neutral competition among stem cell progenies, gives rise to distinct DNA methylation patterns between crypts [31, 32]. In 2007, Nicolas et al. used Bayesian modeling of DNA methylation data to infer that each crypt contains 15–20 stem cells, and a single stem cell requires 15–40 years to dominate an entire crypt [33]. However, in 2011, Graham et al. labeled clones by mitochondrial cytochrome c oxidase (CCO) mutations and found that although small CCO‐deficient (CCO^−^) clones maintained relatively consistent DNA methylation patterns, larger clones exhibited rapid DNA methylation divergence, suggesting that DNA methylation can only trace lineage relationships over 10–20 years and may not be suitable for longer timescales [34]. Overall, these studies indicated that DNA methylation patterns appear to be a powerful “lineage recorder” in the colon that can be used to infer the number of stem cells in the crypt [35], intra‐crypt stem cell competitions, and clonal evolution of individual crypts in the colon over a decade or more [36]. However, these results are not independently validated by other lineage tracing tools and rely only on bulk DNA methylation, which may not generalize to single‐cell analysis.

More evidence comes from cancer research (Figure 2). It is possible to sample cancer cells from multiple regions within a tissue of a donor and infer their evolutionary histories through copy number variations identified with bulk whole‐genome sequencing. Joint profiling of bulk DNA methylation was conducted in several studies, revealing that samples with closer lineages (e.g., with similar CNVs) also had similar DNA methylation profiles [37, 38]. These findings were further consolidated by applying single‐cell multi‐omic assays to study colorectal, gastric, and high‐grade plasmacytoid ovarian cancers, where unsupervised clusters derived from single‐cell DNA methylome were consistent with clones inferred by CNVs [39, 40, 41]. In 2019, Gaiti and others proposed that the phylogenetic tree based on DNA methylation epimutations could reflect the evolutionary history of cancer cells and applied their framework to reconstruct single‐cell lineages for lymphocytic leukemia and glioma [42, 43]. Despite the conceptual innovation, their studies lacked the support from neutral lineage markers to directly validate the inferred lineage trees. Overall, mounting evidence in cancer research indicates the possibility of using DNA methylation to track cancer lineages. However, cancer cells are special due to their unstable genome and epigenome, making it challenging to generalize these results to normal contexts.

DNA methylation also appears to record cell lineages in early embryo development (Figure 2). In 2016, Mooijman et al. developed a single‐cell profiling method for 5hmC, which is an intermediate product during the active demethylation of 5mC [44]. Because 5hmC undergoes passive dilution and asymmetric distribution in preimplantation blastomers, they found that its chromosome‐level pattern could reconstruct the lineage of 2‐cell and 4‐cell mouse embryos. Inspired by this work and noting that 5mC also undergoes passive dilution and asymmetric distribution in the first few divisions, Zhu et al. hypothesized that the chromosome‐level pattern of 5mC could also enable lineage tracking in early embryo development [45]. Indeed, they demonstrated that 5mC also discriminated the cell lineages during the 4‐cell and 8‐cell stages [45, 46]. However, these successes rely on passive dilution and asymmetric distribution of DNA methylation during cell division, which cannot be generalized to other contexts.

Unlike mammals, DNA methylation in plants does not undergo global demethylation between generations. This enables the accumulation of epimutations over multiple generations that span hundreds of years. In 2023, Yao et al. utilized this feature to develop an evolutionary epigenetic clock in plants based on bulk DNA methylation sequencing of plant tissues [47]. Applying this to *Arabidopsis thaliana*, they demonstrated that these clock‐like epimutations successfully recapitulate known phylogenies of these trees within a very recent timescale (Figure 2).

Finally, our recent work demonstrates strong methylation‐related clonal memory in hematopoietic stem cells (HSCs) in normal mice (Figure 2). In 2023, taking advantage of our lineage tracing mouse model DARLIN, we labeled HSCs in vivo at E10 and waited until either E15.5 or the adult stage to profile HSCs with Camellia‐seq, which can simultaneously profile DNA methylation, transcriptome, chromatin accessibility, and lineage barcode for each cell [7]. To our surprise, we found that cells from the same clone share a more similar DNA methylation pattern, despite the month‐long labeling that is accompanied by HSC migration and massive proliferation. In contrast, transcriptome and chromatin accessibility could not distinguish cells from different clonal origins. Our study provides the first direct evidence at the single‐cell level that the DNA methylome robustly records clonal memory in vivo over a long time.

## 3 METHYLTREE: THE FIRST GENERIC LINEAGE‐TRACING TOOL BASED ON EPIMUTATIONS

Although these studies suggest that DNA methylation could track clonal relationships, it remains unclear how accurate this could be and whether this would work generally throughout differentiation and across different biological contexts. To solve this problem, we must tackle four key challenges. First, single‐cell DNA methylation data are highly sparse, with just about 5% genomic coverage per cell, making it challenging to accurately capture cellular differences [48]. Second, the technical noise introduced during library construction and sequencing could interfere with the extraction of lineage signals [48, 49]. In addition, numerous studies have shown that DNA methylation regulates gene expression, leading to strong cell‐type‐specific signals, making it difficult to separate functional methylation differences from epimutations [50, 51]. Lastly, the global DNA methylation level is heavily modulated during development, which may erase lineage signals [52, 53, 54, 55].

Although Gaiti et al. built the first single‐cell lineage tree from DNA methylation in blood cancers, they did not address the above challenges directly [42]. They utilized IQ‐Tree, a well‐established method for constructing phylogenetic trees in the field of evolutionary studies, to build a lineage tree from sparse single‐cell DNA methylation data. IQ‐Tree has a built‐in approach to handle missing values. However, it is computationally highly expensive and can take many hours to build trees for just 100 cells with 29 million CpG sites. More importantly, Gaiti et al. did not explicitly address the problem of cell‐type‐related methylation signals. Due to these issues, their approach cannot be applied to systems with cell differentiation. However, studying cell fate choice during differentiation is a major goal for developing lineage tracing tools.

**FIGURE 3:**
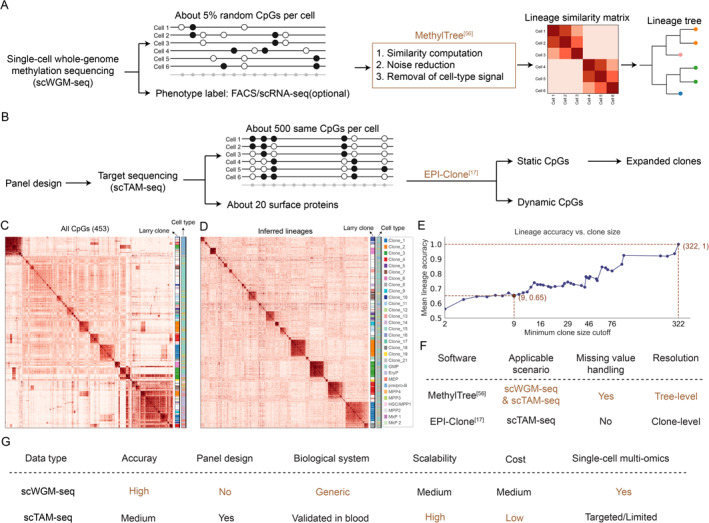
Comparison between MethylTree and EPI‐Clone. (A) Workflow of MethylTree. Sparse single‐cell DNA methylome data are obtained from single‐cell whole‐genome methylation sequencing (scWGM‐seq), with optional phenotypic labels from fluorescence‐activated cell sorting (FACS) and single‐cell RNA sequencing (scRNA‐seq). MethylTree can be applied to compute cell‐cell similarity, correct noise, and remove cell‐type signals. The lineage tree can be inferred from the resulting lineage similarity matrix. (B) Schematic of EPI‐Clone workflow, which includes CpG panel selection, targeted sequencing, and data analysis. Crucially, static CpG sites are identified with EPI‐Clone from the CpG panels to enable the identification of expanded clones. (C, D) Lineage reconstruction by MethylTree on the EPI‐Clone dataset. (C) Heatmap of the raw similarity matrix computed using all the approximately 500 CpGs in the data. (D) Heatmap of lineage similarity after removing cell type signals in (C) with the built‐in MethylTree method. (E) Lineage tracing accuracy of MethylTree on the EPI‐Clone dataset across different minimum clone size thresholds, after removing cell type signals. Clone size 9 is the threshold for defining expanded clones in EPI‐Clone [17]. In MethylTree, using all 453 CpGs from EPI‐Clone, the lineage accuracy reaches 0.65 after removing cell‐type signals. Accuracy is defined as the mean proportion of cells in the largest contiguous segment within each clone in the similarity matrix, as defined in the MethylTree paper. 322 is the size of the largest clone in this data. (F) Software‐level comparison between MethylTree and EPI‐Clone. MethylTree supports broader applications, offers higher resolution, and handles missing data. (G) Comparison of lineage tracing based on either scWGM‐seq (genome‐wide approach) or scTAM‐seq (targeted approach).

In January 2025, we published MethylTree, which successfully overcame the above four key challenges [56, 57]. Specifically, MethylTree utilizes jointly observed CpG sites between two cells to calculate their Pearson correlation coefficients (Figure 3A). This addresses the issue of massive missing data and gives a correlation matrix that reflects the lineage similarity of these cells. To deal with heterogeneous noises, MethylTree iteratively infers a noise factor for each cell and corrects the similarity matrix accordingly. In general, we found that MethylTree performs much better after performing this noise‐cleaning. Finally, based on prior cell type information, which could come from either fluorescence‐activated cell sorting (FACS) or joint transcriptome profiling, MethylTree can remove functional methylation signals related to different cell types, thereby extracting lineage‐specific similarity for downstream analysis (Figure 3A). With these approaches, MethylTree reconstructed the cellular lineages from single‐cell DNA methylomes with an accuracy rate of around 100% across broad biological conditions. This makes MethylTree the first generic lineage tracing tool based on single‐cell DNA methylation.

The most convincing validation of MethylTree came from in vitro differentiation experiments with HSCs. After isolating HSCs from mice, progenitor cells of each clone were uniquely labeled by LARRY lentivirus and induced to differentiate in vitro. The LARRY lentivirus added lineage‐specific barcodes to each founder cell, which can be read out later as the ground‐truth lineage barcodes that were used to benchmark MethylTree. On day 6, cells were collected to simultaneously profile the transcriptome, DNA methylome, and LARRY lineage barcode in each cell. This gave 52 multicellular clones, 21 of which comprised multiple cell types. MethylTree successfully distinguished all clones based on DNA methylation with 100% accuracy. The same results were obtained in the human hematopoietic system: after isolating HSCs from umbilical cord blood, labeling them with LARRY, and culturing them in vitro for 13 days, MethylTree also identified 20 multicellular clones with 100% accuracy, 9 of which contained multiple cell types. In addition to the hematopoietic system, MethylTree has been validated in a variety of systems, including human and mouse embryonic development, the in vivo HSCs of the DARLIN mouse, as well as multiple cell lines (e.g., 293T and H9) and colon cancer. These results demonstrate that DNA methylation is a stable and high‐resolution “lineage recorder” and MethylTree can accurately and noninvasively resolve single‐cell lineages.

Applying MethylTree, we discovered stochastic early fate commitment at the 4‐cell stage in human embryo development, which is consistent with reports coming out at the same time. In addition, we estimated for the first time that mouse bone marrow has roughly 250 clones of HSCs, corresponding to around 250 de novo HSCs generated during endothelial‐to‐hematopoietic transitions.

## 4 EPI‐CLONE: A COMPLEMENTARY APPROACH WITH TARGETED SEQUENCING

A complementary approach called EPI‐Clone (Figure 3B) was published 4 months after MethylTree [17]. Although MethylTree used single‐cell whole‐genome methylation sequencing (scWGM‐seq), EPI‐Clone is based on targeted single‐cell DNA methylation sequencing. Specifically, this group previously developed scTAM‐seq, a scalable and targeted methylation sequencing method that can profile the methylation status of several hundred CpGs in single cells using the Mission Bio Tapestri platform. scTAM‐seq achieves efficient readout across cells at the same CpG sites, with a low dropout rate of just 7% [58]. The conceptual innovation here is to design a set of around 500 targeted CpGs, including both cell‐type‐specific CpGs (so‐called dynamic CpGs) as well as functionally neutral CpGs that could undergo epimutations and, therefore, record cell lineages (static CpGs). Designing this CpG panel is the major challenge here. The authors obtained such a tentative list by mining previously published bulk whole‐genome DNA methylation data from different cell types. Using this CpG panel, a scTAM‐seq dataset was generated along with the expression of approximately 20 surface protein markers for each cell. Then, the EPI‐Clone algorithm was applied to this dataset to identify both static and dynamic CpGs from this panel. EPI‐Clone essentially performs a statistical test on this data to identify CpGs that correlate with differential expression of protein markers as dynamic CpGs, and assigns CpGs that do not have such correlation as static CpGs. Finally, cells can be clustered into different clones in the low‐dimensional space generated with the static CpGs (Figure 3B). Note that EPI‐Clone can only identify expanded clones and has low resolution with small clones.

Similar to how we validated MethylTree, Scherer et al. used LARRY barcodes to benchmark the performance of EPI‐Clone. Here, they transplanted LARRY‐barcoded HSCs to host mice, waited several months, and profiled their progenies with scTAM‐seq, along with LARRY barcodes and surface protein expression for each cell. Applying EPI‐Clone, their predicted expanded clones achieved an accuracy of around 0.8 in terms of adjusted rank index or ARI. This is actually quite impressive, given that they only used around 500 CpG sites. Furthermore, they applied EPI‐Clone to study blood aging in both mice and humans, and identified an increasing number of expanded clones in both cases.

## 5 METHYLTREE WORKS FOR scTAM‐SEQ DATA

MethylTree is an out‐of‐the‐box computational package that can infer cell lineages from single‐cell DNA methylation data, whether it is from whole‐genome sequencing or targeted amplification. Therefore, MethylTree should also work for scTAM‐seq data. To demonstrate this, we applied MethylTree to one of the LARRY datasets generated in the EPI‐Clone paper. Before removing the cell‐type signal, we can see that the MethylTree similarity matrix contains both cell‐type and lineage signals (Figure 3C). However, after removing the cell‐type signal, we observed strong lineage signals that correlate well with the LARRY barcodes (Figure 3D). Consistent with the performance of EPI‐Clone, MethylTree performed well in identifying large clones in this targeted dataset, but failed to detect small clones (Figure 3E). This is likely due to having just around 500 CpG sites. Besides, the 7% dropout per CpG site in the scTAM‐seq measurement may also partially contribute to this noise. Here, unlike the scenario in scWGM‐seq, these dropout events cannot be distinguished from the real “unmethylated” state in scTAM‐seq, leading to noise. Therefore, MethylTree provides a unified solution for inferring lineages from different single‐cell DNA methylation data.

## 6 METHYLTREE VS EPI‐CLONE: A COMPARISON

First, we take a narrower view of EPI‐Clone as a computational method that separates static and dynamic CpGs and infers expanded clones from a low‐dimensional embedding of static CpGs. Similarly, we consider MethylTree also as a computational method for lineage prediction from DNA methylation data. In this narrower view, MethylTree is a more sophisticated computational tool that works with both genome‐wide approaches like single‐cell whole‐genome methylation sequencing (scWGM‐seq), and targeted approaches such as scTAM‐seq, as we demonstrated above. However, EPI‐Clone can only work with scTAM‐seq, as it cannot handle missing values in scWGM‐seq. In addition, MethylTree cleans up data noise and infers phylogenetic trees, while EPI‐Clone relies on dimension reduction and clustering, predicting only expanded clones. Furthermore, unlike EPI‐Clone, MethylTree does not require the identification of static CpGs for lineage tracing, making it simpler and potentially more robust than EPI‐Clone. Therefore, in terms of data analysis, MethylTree will be the preferred method for inferring cell lineages from single‐cell DNA methylation data (Figure 3F).

In a broader view, MethylTree represents the genome‐wide and omics approach (Figure 3A), whereas EPI‐Clone represents the targeted method based on scTAM‐seq (Figure 3B). These two approaches are certainly complementary to each other. However, they also have important differences. The targeted approach has the advantage of being scalable and cost‐effective. In the EPI‐Clone paper, tens of thousands of cells have been profiled with scTAM‐seq. On the other hand, scWGM‐seq currently has a lower throughput and higher cost. The throughput is not an inherent issue, since several recent studies have generated whole‐genome DNA methylomes for tens of thousands of cells [59, 60, 61, 62]. However, whole‐genome sequencing is definitely more costly.

The genome‐wide approach works directly across different biological systems, whereas the targeted approach requires careful CpG panel selection when studying a new tissue. Indeed, MethylTree has been demonstrated to have approximately 100% accuracy across a wide range of cell types and developmental stages, while EPI‐Clone has been validated only in blood. Once good CpG panels with annotated static and dynamic CpGs are established and validated for a given tissue, the targeted approach is an appealing option. However, establishing these CpGs for a new tissue would be challenging because the current EPI‐Clone pipeline requires jointly detected surface protein expression to resolve static and dynamic CpGs. Most human tissues do not have well‐annotated surface protein markers for different cell types. Therefore, beyond blood, the genome‐wide approach and MethylTree would be the preferred option.

Furthermore, with the genome‐wide approach, MethylTree has the advantage of higher accuracy and resolution by exploiting all the approximately 29 million CpG sites across the genome. This is in comparison to the approximately 500 CpG sites generated in scTAM‐seq. Indeed, EPI‐Clone currently only identifies large expanded clones, whereas MethylTree achieved 100% accuracy for both large and small clones. Furthermore, MethylTree not only resolves clones but also produces phylogenetic trees that have higher temporal resolution. These are demonstrated with in vitro single‐cell expansion of HEK293T cells and also in the application of human early embryo development, where MethylTree resolved the division histories within a single developing embryo.

Finally, although the targeted scTAM‐seq data is tailored specifically for lineage tracing, the genome‐wide measurements can be useful for not only lineage tracing, but also exploratory analysis of the role of epigenome (i.e., DNA methylome here) in cell fate choice. Many methods exist to jointly profile single‐cell DNA methylome with other modalities, such as transcriptome, chromatin accessibility, and 3‐D chromatin architecture [7, 59, 60, 62]. Therefore, MethylTree natively supports single‐cell multi‐omic lineage tracing in humans, which enables systematic and data‐driven dissection of lineage dynamics in humans. In comparison, the targeted approach is more limited in exploratory analysis.

In summary, the genome‐wide and targeted approaches are complementary to each other, and both have their merits. The targeted approach is more scalable and cost‐effective, whereas the genome‐wide approach has higher accuracy and richer information for exploration. So far, targeted CpG panels have been established only in blood, making the genome‐wide approach the preferred option for studying other tissues. Importantly, MethylTree works robustly with both the genome‐wide and targeted approaches, thus providing a unified framework for lineage analysis with DNA methylation data (Figure 3G).

## 7 HOW TO USE METHYLTREE

MethylTree is a well‐structured and user‐friendly Python package that enables methylation‐based lineage tracing across broad biological contexts on GitHub website (ShouWenWang‐Lab/MethylTree). To use MethylTree for lineage tracing, one would generally need to obtain the single‐cell DNA methylome data first, along with the phenotypic label for each cell. Such a phenotypic label could be obtained from FACS sorting, the corresponding single‐cell transcriptome, or other means. The phenotypic information is needed to regress out the cell‐type‐specific DNA methylation information. For more information, please visit the GitHub website (ShouWenWang‐Lab/MethylTree_notebooks).

## 8 EPIMUTATION‐BASED LINEAGE TRACING: ADVANTAGES AND FUTURE DEVELOPMENT

So far, noninvasive lineage tracing in humans can be achieved with mutations in nuclear DNA, mitochondrial DNA, or epimutations in DNA methylation. However, as mentioned in the introduction, the inheritance of mitochondrial DNA mutations is complex, and the inferred lineage is often inaccurate; nuclear DNA accumulates just about 1 mutation per division, which is very difficult and costly to measure. Indeed, it costs around $500 for the combined cost of clonal expansion, library generation, and deep sequencing. Besides, currently it does not support simultaneous profiling of other modalities, such as transcriptome and epigenome in single cells.

**FIGURE 4:**
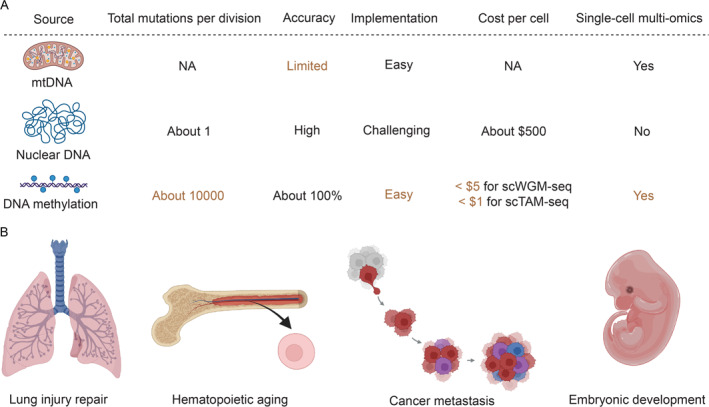
Advantages and applications of epimutation‐based lineage tracing. (A) Comparison between three endogenous lineage tracing sources: mtDNA mutation, nuclear DNA mutation, and epimutation on DNA methylation. (B) Possible biological applications.

In contrast, DNA methylation accumulates approximately 10,000 epimutations per cell division and can be easily measured at single‐cell resolution, making it an ideal lineage recorder. Furthermore, single‐cell DNA methylome can be jointly profiled with other modalities such as transcriptome. When integrated with MethylTree, this would enable single‐cell multi‐omic lineage tracing to systematically dissect development and disease in humans. Currently, at the sequencing depth needed for MethylTree (i.e., around 5% genomic coverage), the cost of such single‐cell multi‐omics profiling has dropped to about $5 per cell, including both library construction and sequencing, which is only around 1% of the cost of somatic‐mutation‐based lineage tracing. With a targeted approach such as scTAM‐seq, the cost could drop much further. Thus, MethylTree provides a practical solution for cost‐effective, high‐resolution, and noninvasive lineage tracing in humans (Figure 4A).

This is only the beginning of epimutation‐based lineage tracing, and much remains to be improved in the near future. At the moment, although scWGM‐seq provides rich information and high resolution, it is costly and has a relatively low throughput. On the other hand, scTAM‐seq is cost‐effective and scalable, but captures only limited information from around 500 CpG sites. In the future, it would be useful to develop an epimutation‐based lineage tracing platform that achieves a good balance between accuracy, scalability, and cost. Developing a more scalable scWGM‐seq approach that can also capture other modalities has its own merit. An improved targeted approach would also be very appealing, especially if it can capture more CpG sites to provide higher resolution. Although epimutation should, in principle, provide rich phylogenetic information, currently the validation largely stays at the clone level in both MethylTree and EPI‐Clone, by using LARRY‐based barcodes for an orthogonal benchmark. Directly validating the phylogenetic resolution could be carried out using serial single‐cell expansion experiments, with an experimental design that is more sophisticated than what we did with HEK293T cell lines in MethylTree. Furthermore, it would be valuable to computationally extract further information from single‐cell DNA methylation, such as cell division time. Finally, adding a spatial modality, although technically challenging, would be an exciting future direction to enable spatial lineage tracing in humans.

## 9 BIOLOGICAL APPLICATIONS AND CONSIDERATIONS

Epimutation‐based lineage tracing, as exemplified by MethylTree and EPI‐Clone, opens the door for single‐cell multi‐omic lineage tracing in humans and other species (Figure 4B). It enables the joint study of cell lineages, transcriptome, and DNA methylome in primary tissues at the single‐cell level. It would greatly facilitate the study of cell lineages in diverse problems in humans, such as embryonic development, organogenesis, cancer evolution, and tissue regeneration. This approach should work across many different tissues, such as blood, lung and intestine. However, DNA methylation is an important layer of epigenetic regulation that could undergo drastic modulation in certain biological processes, such as cancer metastasis, immune cell activation, or germ cell development. In addition, nondividing cells such as neurons could accumulate many epigenetic changes over their lifetime, which may create excessive noise in lineage reconstruction. Despite the early success of MethylTree in resolving the lineage of these biological processes, more research is needed to understand these processes better and develop a more robust lineage‐tracing strategy in these special contexts. Despite these caveats, epimutation‐based lineage tracing should create an exciting moment for studying cell fate choice in human tissues across many biological contexts.

## AUTHOR CONTRIBUTIONS

**Ruijiang Fu**: Writing—original draft; Writing—review and editing; visualization. **Mengyang Chen**: Writing—review and editing. **Shou‐Wen Wang**: Project administration; supervision; funding acquisition; writing—review and editing.

## CONFLICT OF INTEREST STATEMENT

The authors declare no conflicts of interest.

## ETHICS STATEMENT

This is a review article and does not contain any studies with human or animal subjects performed by any of the authors.

## Data Availability

This work does not produce any new data.
